# Periaqueductal/periventricular gray deep brain stimulation for the treatment of neuropathic facial pain

**DOI:** 10.3389/fneur.2023.1239092

**Published:** 2023-11-07

**Authors:** Victor Mandat, Pawel R. Zdunek, Bartosz Krolicki, Krzysztof Szalecki, Henryk M. Koziara, Konrad Ciecierski, Tomasz S. Mandat

**Affiliations:** ^1^Department of Neurosurgery, Maria Sklodowska-Curie National Research Institute of Oncology, Warsaw, Poland; ^2^Department of Biology, University of Toronto, Toronto, ON, Canada; ^3^Research and Academic Computer Network Organization (NASK), Warsaw, Poland

**Keywords:** periaqueductal gray, periventricular gray, deep brain stimulation, neuropathic pain, face

## Abstract

**Background:**

The Periaqueductal gray (PAG) and the periventricular gray (PVG) are the anatomical targets for deep brain stimulation (DBS) to treat severe, refractory neuropathic pain.

**Methods:**

Seven (four female and three male) patients were qualified for PAG/PVG DBS because of neuropathic facial pain. Frame-based unilateral implantations of DBS were conducted according to indirect planning of the PAG/PVG, contralateral to reported pain (3389, Activa SC 37603, Medtronic). The efficacy of PAG/PVG DBS on pain was measured with Numeric Pain Rating Scale (NRS) and Neuropathic Pain Symptom Inventory (NPSI) before surgery and 3, 12, and 24 months after surgery.

**Results:**

The mean age of the group at the implantation was 43.7 years (range: 28–62; SD: 12.13). The mean duration of pain varied from 2 to 12 years (mean: 7.3; SD: 4.11). Five patients suffered from left-sided facial pain and two suffered right-sided facial pain. The etiology of pain among four patients was connected to ischemic brain stroke and in one patient to cerebral hemorrhagic stroke. Patients did not suffer from any other chronic medical condition The beginnings of ailments among two patients were related to craniofacial injury. NRS decreased by 54% at the 3 months follow-up. The efficacy of the treatment measured with mean NRS decreased at one-year follow-up to 48% and to 45% at 24 months follow-up. The efficacy of the treatment measured with NPSI decreased from 0.27 to 0.17 at 2 years follow-up (mean reduction by 38%). The most significant improvement was recorded in the first section of NPSI (Q1: burning- reduced by 53%). The records of the last section (number five) of the NPSI (paresthesia/dysesthesia- Q11/Q12) have shown aggravation of those symptoms by 10% at the two-years follow-up. No surgery- or hardware-related complications were reported in the group. Transient adverse effects related to the stimulation were eliminated during the programming sessions.

**Conclusion:**

PAG/PVG DBS is an effective and safe method of treatment of medically refractory neuropathic facial pain. The effectiveness of the treatment tends to decrease at 2 years follow-up. The clinical symptoms which tend to respond the best is burning pain. Symptoms like paresthesia and dysesthesia might increase after DBS treatment, even without active stimulation.

## Introduction

Chronic pain is a complex phenomenon described by Spinoza as a “localized form of sorrow.” Neuropathic pain appears as a result of central, peripheral or autonomic nervous system damage. Neuropathic pain is undulating and persistent. Patients usually described neuropathic pain as a constant, burning sensation, but clinical phenotype might vary and various forms of attacks may additionally be present. Pain involves multiple neuronal circuits. The elementary understanding of neuropathic pain focuses on lateral pain pathways. The nociceptive stimuli are carried by A-delta and C fibers to the dorsal root ganglions, spinothalamic tracts and through the thalamus to the postcentral gyrus (1, 2). When the pain persists, an affective component of the neuropathic pain phenomenon becomes more significant. With the increased components of the affective and limbic systems, pain becomes less localized and discriminated (3). The limbic pathway projects to the thalamus, hippocampus, cingulate and nucleus accumbens. Involvement of affective and limbic pathways, with time impairs more significantly the quality of patients’ life (4). Target treatments toward the limbic system from somatosensory pathways might result in significant improvement in neuropathic pain perception. In parallel to limbic, affective and somatosensory pathways, the cognitive functions construct the fourth pillar of neuropathic pain perception (2–4). Standard treatment is focused on pharmacotherapy (analgesic ladder) that includes antiepileptic and mood-enhancing medications. Psychological support is one of the key pillars of the treatment. Appropriate physiotherapy improves the results of the treatment. Surgical treatment might be considered if the effects of conservative treatment are unsatisfactory (5–7).

Deep brain stimulation (DBS) is a well-established method of neurosurgical treatment for movement disorders, especially: Parkinson’s disease, essential tremor and dystonia (7, 8). Initial attempts to treat surgically movement disorders were undertaken in the first half of the twentieth century. Electric stimulation was used at that time, to test for side effects prior to execution of permanent thermal or chemical lesion of the basal ganglia or midbrain. The first applications of electrical stimulation to the brain prior to the thermal lesions in the treatment of pain were conducted as early as in the 1950s (5). In the 1980’s with the introduction of modern DBS hardware and software with FDA approval, a renaissance of functional neurosurgery has begun. DBS has a better side-effect profile compared to ablative procedures (9–11). The stimulation parameters are adjustable and possible side effects are reversible. It is believed that functional neurosurgery is the fastest-growing supraspecialisation in neurosurgery today (8). It is estimated that almost two hundred thousand patients were implanted with DBS worldwide until 2022. The number of research papers linked to DBS surpassed 1,000 publications annually a decade ago. The possible applications for DBS, not only in neurological disorders are constantly expanding. DBS has been approved for pain, medically refractory epilepsy and psychosurgery. Through its reversible action, DBS has become an effective and invaluable preclinical and clinical research tool. With the application of DBS in laboratory models, neural networks are better understood today (10, 12, 13).

The periaqueductal gray (PAG) and the periventricular gray (PVG) were predominantly identified as the anatomical target for nociceptive pain, whereas the thalamus was aimed to treat neuropathic pain. The role of PAG/PVG in the pain regulation process can not be overrated. Ascending, nociceptive afferents run through PAG/PVG to the thalamus. In the other direction, reciprocal regulatory control of the PAG/PVG modulates the dorsal horns of the spinal cord activation following peripheral nociceptive stimuli. Stimulation of central gray matter of the tegmentum and midbrain (PAG/PVG) by inhibiting nociceptive responses and increasing endogenous opioid levels is believed to be effective for neurogenic pain. The effect of PAG/PVG DBS is pharmacologically reversible with the application of opioid antagonists (14–16). It is not clear if increased levels of endogenous opioids are related to direct or indirect stimulation of PAG/PVG (17–19). Mostly because of the size of PAG/PVG and the proximity of surrounding structures its sensing topography has not been described yet and requires further exploration. The role of PAG/PVG stimulation in the regulatory process of autonomic functions and mood alterations like fear have been described (17, 19).

There is a noticeable deficit of data about the mechanism of action of PAG/PVG DBS on neuropathic pain. The authors believe that the presented analysis will allow for a better understanding of the mechanism of action of PAG/PVG DBS on pain and will lead to further research. The objective of the study was to evaluate the effectiveness and safety of PAG/PVG DBS on neuropathic facial pain. The authors aimed to identify the most optimal clinical characteristics of pain for this invasive but reversible treatment.

## Methods

**Figure 1:**
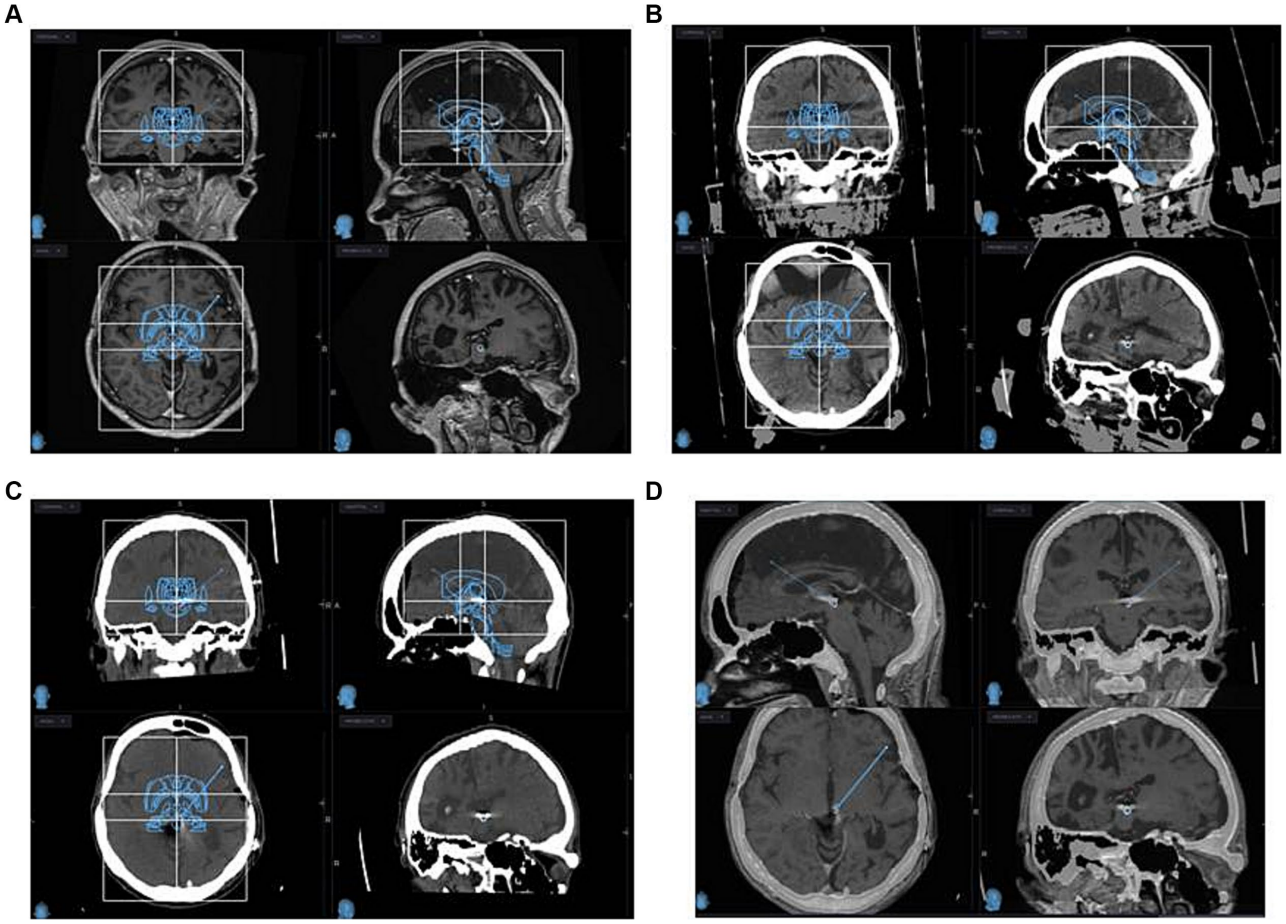
MRI **(A)** and CT with stereotactic frame **(B)** planning images of PAG/PVG DBS implantation are depicted. Post-implantation CT **(C)** and MRI **(D)** images are depicted. Infused Schaltenbrand Wahren atlas and Talairach grids are visualized for indirect identification of PAG-PVG **(A–C)** at coronal, sagittal, and axial projections.

Seven eligible patients were diagnosed with neuropathic facial pain according to the IASP Neuropathic Pain Special Interest Group (NeuPSIG) definition. Those patients were qualified by pain specialists, psychologists and neurosurgeons for PAG/PVG DBS. None of the patients had a history of psychiatric treatment. Patients underwent a standard battery of psychological tests performed for qualification for neuromodulation pain treatment. None of the patients suffered from major depression. Patients had psychological support before and after surgical treatment. All patients qualified for the surgery underwent typical conservative treatment including an analgesic ladder and adjuvant therapy that was ineffective (8, 12). No chronic comorbidities were reported. Consent forms were obtained from the patients before each interview. Ethics committee approval was not required as long as the patients underwent standard treatment and evaluation that included examining patients for co-morbidities, surgery and other non-invasive medical assessments. Demographic data were collected and analyzed: initials, age (date of birth), gender, disease duration and clinical status measured with the Numeric Pain Rating Scale (NRS) and the Neuropathic Pain Symptom Inventory (NPSI) of four male and three female patients (3, 6, 20). NPSI consists of 12 questions and five sub-scores (Q1- burning, Q2 + 3- pressing, Q5 + 6 paroxysmal pain, Q8 + 9 + 10- paroxysmal pain, Q11 + 12- paresthesia/dysesthesia). Additional questions: Q4 and Q7 represent overtime nuisance. The calculated total intensity pain score measured with NPSI consists of five sub-scores multiplied by two and divided by 100% (6, 20). The efficacy of PAG/PVG DBS on facial pain was measured with NRS and NPSI before surgery, 3, 12, and 24 months after surgery. Frame-based unilateral implantation of DBS was conducted according to indirect planning of the PAG/PVG contralateral to reported pain (Figure 1). The anatomical target of PAG/PVG DBS is parallel to the midbrain aqueduct. The stereotactic coordinates for PAG/PVG were: 2–3 mm lateral from the wall of the third ventricle, 1–2 mm anterior to the posterior commissure at the level of the intercommiusural lane. The entry point was located one to three centimeters in front of the coronal suture and four to five centimeters lateral from the midline. The patients were sedated during the surgical procedure and no neurophysiological evaluations were conducted during surgery. All patients were implanted with 3,389 brain electrodes and Activa SC37603 internal pulse generator (Medtronic). On the day following implantation, a control brain CT was performed and the positions of the electrodes were confirmed with the presurgical planning (Figure 1). The stimulation was initialized on the day following implantation. The initial settings were: monopolar stimulation (case positive, contact 1 and 2 negative) with amplitude 1 V, frequency 50 Hz, and pulse width 60 μs. Depending on clinical response, the amplitude was gradually increased up to 5 V. The frequency and pulse width remained unchanged in the analyzed group. The main outcome measures were the NRS and NPSI scores. Possible adverse effects were recorded. The interviews for the pain evaluation and adverse effects were carried out at the outpatient clinic 3, 12, and 24 months after implantation. The statistical analysis was conducted with Statistica 8.0 PL and the graphs were prepared with MS Excel (Microsoft Corporation).

## Results

**Figure 2:**
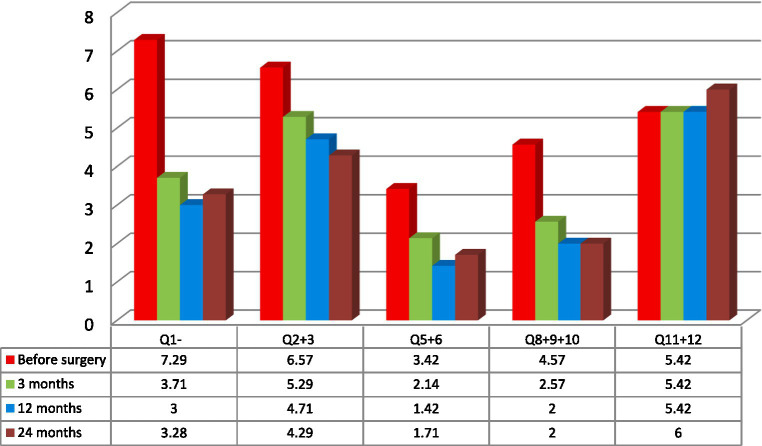
Mean variations of spontaneous pain measured with Neuropathic Pain Symptom Inventory (NPSI) subscales measured in the analyzed group of seven patients: Q1- burning (superficial); Q2- pressure and Q3 squeezing (deep); Q5- electric shocks and Q6- stabbing (paroxysmal); Q8- evoked by brushing, Q9- evoked by pressure and Q10- evoked by cold stimuli (evoked); Q11- pins and needles, and Q12 tingling (paresthesia/ dysesthesia) (6, 20).

Seven patients completed evaluation before the surgery and after PAG/PVG DBS implantation at the follow-up (3, 12, and 24 months following surgery). The mean age of the group at the implantation was 43.7 years (range: 28–62; SD: 12.13). The mean duration of pain varied from 2 to 12 years (mean: 7.3; SD 4.11). Five patients suffered from left-sided facial pain and two suffered right-sided facial pain. The etiology of pain among four patients was connected to brain ischemic stroke and one patient had a history of brain hemorrhagic stroke. The beginning of ailments among two patients was related to craniofacial injury. Two patients (#2, 3) in the group had a history of motor cortex stimulation that did not influence their pain score or quality of life. The systems were explanted 6 months and 10 months after implantation. Three patients (#1, 4, 7) underwent thalamic DBS without significant effect as well. Those systems were explanted 12, 16, and 21 months after surgery. One patient (#6) with a mixed character of pain (neuropathic facial pain and trigeminal neuralgia) had a history of microvascular decompression and radiofrequency lesions in the Gasserian ganglion that were ineffective. The mean NRS score before surgery was 8.7 (8–10; SD:0.78; Table 1). The mean NPSI score before surgery was 0.27 (0.22–0.4; SD: 0.096; Table 2). NRS decreased by 54% at 3 months follow-up. The efficacy of the treatment measured with NRS decreased at one-year follow-up to 48% and to 45% at 24 months follow-up (Table 1). The total pain intensity score measured with NPSI decreased from 52% before surgery through 38% after 3 months and 32% after 12 months to 34% at the two-years follow-up. The most significant improvement was recorded in the first section of NPSI (Q1: burning- reduced by 53%). The last section of the test (Q11 + 12: paresthesia/ dysesthesia) showed aggravation of the symptoms by 10% at the two-years follow-up (Figure 2).

**Table 1:** Pain Numeric Rating Scale (NRS) score before surgery and 3, 12, and 24 months following surgery measured in the analyzed group of seven patients: (score and percentage of improvement are displayed) (3).

Subject	Before surgery	3 months		12 months		24 months	
	NRS score	NRS	%	NRS	%	NRS	
1	8	4	50%	4	50%	4	50%
2	8	3	63%	3	63%	3	63%
3	8	4	50%	6	25%	8	0%
4	9	4	50%	3	66%	3	66%
5	10	5	50%	5	50%	5	50%
6	8	4	50%	4	50%	4	50%
7	9	3	66%	6	33%	6	33%

**Table 2:** Neuropathic Pain Symptom Inventory (NPSI) subscales score measured before surgery in the analyzed group of seven patients: Subscales: Q1- burning (superficial); Q2- pressure and Q3 squeezing (deep); Q5- electric shocks and Q6- stabbing (paroxysmal); Q8- evoked by brushing, Q9- evoked by pressure and Q10- evoked by cold stimuli (evoked); Q11- pins and needles, and Q12 tingling (paresthesia/dysesthesia), Q4- spontaneous pain during the past 24 h, Q7- pain attack during the past 24 h (6, 20).

Subject	Q1	Q2 + 3	Q5 + 6	Q8 + 9 + 10	Q11 + 12	Q4	Q7
1	8	8	2	0	4	8	10
2	7	4	0	4	6	8	8
3	4	10	6	10	8	10	10
4	8	4	6	0	4	10	6
5	10	8	6	10	10	10	10
6	6	8	2	4	4	8	6
7	8	4	2	4	2	8	4

**Figure 3:**
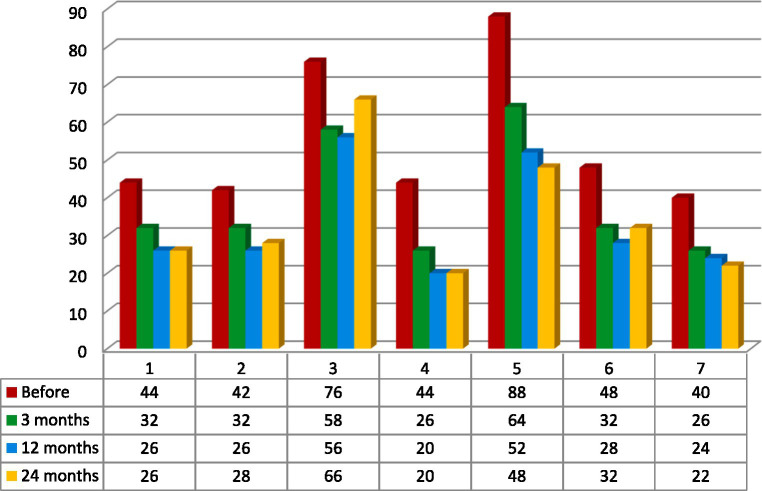
Total intensity pain score. Calculation based on a sum of Neuropathic Pain Symptom Inventory (NPSI) subscales measured in the analyzed group of seven patients: Q1- burning (superficial); Q2- pressure and Q3 squeezing (deep); Q5- electric shocks and Q6- stabbing (paroxysmal); Q8- evoked by brushing, Q9- evoked by pressure and Q10- evoked by cold stimuli (evoked); Q11- pins and needles, and Q12 tingling (paresthesia/dysesthesia) (6, 20).

**Figure 4:**
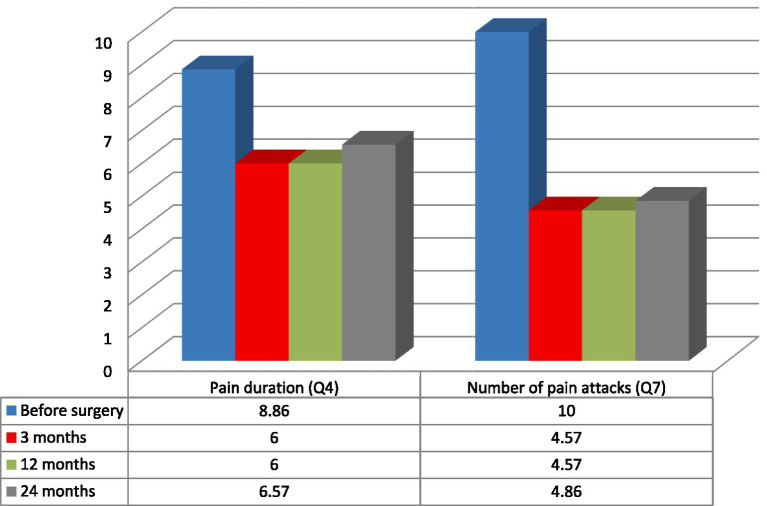
Duration of pain and the number of pain attacks measured with NeuropathicPain Symptom Inventory (NPSI) in the analyzed group of seven patients: mean pain duration (Q4) and the number of pain attacks (Q7) (6, 20).

Patients’ age, sex, previous neuromodulation treatment, duration, etiology and laterality of pain were not prognostic variables for the efficacy of the treatment. Because of the small group of patients and additionally, because of its’ inhomogeneity the results were not statistically significant. The best results were recorded among two patients: 28-year-old male (#2) with 3 years history of poststroke pain (NRS- 63% and NPSI- 43% improvement; Q1–71% improvement and unchanged score in Q2 + 3 subscale) and 36-year-old female (#4) with a 12 years history of posttraumatic pain (NRS- 66% and NPSI 45% improvement; Q1–75% improvement and 20% improvement in Q2 + 3 subscale). The worst results were recorded among two patients: 48-year-old female (#3) with a 12 years history of poststroke pain (NRS- 0% and NSPI- 13% improvement; Q1–25% improvement without change in Q2 + 3) and 48-years-old female (#7) with a 2 years history of posttraumatic pain (NRS- 33% and NPSI 45% improvement) (Tables 1, 3; Figure 2). The total intensity pain score in the whole group of patients decreased in the follow-up. The initial better response of the total intensity pain score recorded in 48-years-old woman (#3) with poststroke pain was reduced (Figure 3). PAG/PVG had a positive effect on overtime nuisance measured with NPSI (Q4 and Q7) (Figure 4). No surgery- or hardware-related complications were reported in the analyzed group. Transient adverse effects related to the stimulation including double vision, contralateral to the stimulation paresthesia and undefined by patients feeling of warmth or cold were eliminated during the same programming session by decreasing the amplitude of the stimulation or changing the polarity of the stimulation. Those adverse effects tend to fade away within seconds after reprogramming of the stimulation.

**Table 3:** Neuropathic Pain Symptom Inventory (NPSI) score (subscale 1–5) measured before surgery and 3, 12, and 24 months following surgery in the analyzed group of seven patients (score and percentage of improvement are displayed) (6, 20).

Subject	Before surgery	3 months		12 months		24 months	
	NPSI	NPSI	%	NPSI	%	NPSI	%
1	0.22	0.16	27%	0.13	41%	0.13	41%
2	0.21	0.16	24%	0.13	38%	0.14	43%
3	0.38	0.29	24%	0.28	26%	0.33	13%
4	0.22	0.13	45%	0.1	54%	0.1	45%
5	0.44	0.32	27%	0.26	41%	0.24	45%
6	0.24	0.16	33%	0.14	42%	0.16	33%
7	0.2	0.13	35%	0.12	40%	0.11	45%

## Discussion

Various types of chronic pain affect 5–20% of the population. One of the main clinical targets of functional neurosurgery in parallel to movement disorders is chronic pain. The etiology of chronic pain qualified for DBS include: poststroke pain, cephalalgia, atypical facial pain, phantom limb pain, brachial plexus avulsion and spinal cord injury. In the presented group patients suffered from neuropathic facial pain whereas its etiology was: brain stroke, brain hemorrhage and craniofacial injury. There were no particular causes of neuropathic pain identified to respond the best to DBS (5, 17, 18). The same results were observed in the analyzed group, the cause of the neuropathic pain had no impact on the success of the treatment.

Potential mechanisms of DBS action include: inhibiting or excitation of neural activity and synaptic filtering. The complexity of the neuronal network affected by DBS adds new variables. The majority of theories analyzing the mechanism of DBS on pain are focused on the immediate (weeks) effects of the stimulation (5, 12). The leading theories indicate that electrical stimulation introduced by DBS restores neural network communication to a more physiological state (8, 10). Electric fields applied to axons surrounding the DBS electrode result in the opening and closing of voltage-gated sodium channels. The effect of DBS on action potentials and controlled release of neurotransmitters and the role of synaptic and neural plasticity at the long-term follow-up remain unclear (14–16, 21). Additionally, there is evidence of neurogenesis and synaptogenesis following DBS in animal models. In parallel to the effect of DBS on neurons, there has been shown also an effect on glial cells that alters the surrounding neurochemical environment and neurons (7, 8).

Three primary anatomical targets are identified for pain treatment with DBS: PAG/PVG, sensory thalamus (ventral posterior lateral or medial nuclei) and anterior cingulate cortex. The secondary targets for DBS include the posterior hypothalamus, centro-median-parafascicular complex, ventral and anterior limb of the internal capsule (8, 9, 17). As an alternative to DBS for: poststroke and posttraumatic pain, facial neuropathic pain, phantom limb pain, brachial plexus avulsion and complex regional pain syndrome is motor cortex stimulation (22, 23). Previously published data indicates that PAG/PVG DBS is particularly effective among patients with nociceptive pain whereas patients with neuropathic pain might benefit more from combined PAG/PVG and thalamic DBS. PAG is an area of gray matter located around the midbrain aqueduct. This structure ascends until reaches the third ventricle anteriorly and becomes PVG. PAG/PVG is involved in the coordination of behavioral and autonomic responses, especially pain. PAG/PVG integrates inputs from nociceptive and autonomic afferents, prefrontal cortex, amygdala, reticular formation and hypothalamus (1, 24). Recent studies demonstrated improvement following PAG/PVG DBS in autonomic functions, like cardiovascular (hyper and hypotension), lung function, bladder capacity and motor systems. In parallel PAG/PVG modulates nociceptive signaling to the brainstem and hypothalamus. It is believed that the effect of PAG/PVG DBS is intensified by engaging endogenous opioid-releasing neurons and by inhibiting or altering nociceptive stimulation (15, 22, 23). In the analyzed group the anatomical functions of the patients who underwent DBS implantation were not analyzed. In the analyzed group, all patients with neuropathic pain were qualified for PAG/PVG DBS. Two patients in the group had a prior history of motor cortex stimulation that did not influence their pain score. Three patients underwent thalamic DBS without significant effect as well. The mechanism of DBS on pain despite enormous progress is still not well understood (2, 16, 25).

The benchmark for good response and clinical usefulness for pain relief of neuromodulation is marked for 50% of pain reduction (3, 6, 20). The efficacy of PAG/PVG DBS for neuropathic pain related to stroke, trauma or amputation is estimated to be ~52% of good to excellent response (>50% improvement). It is estimated that 26% of patients will not benefit from the surgery or will respond poorly to the stimulation (<20% improvement). It has to be kept in mind that patients treated with PAG/PVG DBS are refractory to all other forms of treatment and even mild improvement can significantly improve their quality of life (18, 26). The initial improvement measured with NRS following the stimulation in the analyzed group reached 50%. This beneficial effect decreased during 24 months of follow-up. Despite modification of the stimulation parameters and increased voltage of the stimulation, the beneficial effect of PAG/PVG decreased in the follow-up. Fading-away effect of the stimulation in the analyzed group is not clear. The affective and cognitive component of pain related to expectations following complex brain surgery, might play an important role in this vanishing effect where the initial, beneficial effect was altered. The effect measured with total intensity pain score (NPSI) was not that significant. More accurate analysis showed NPSI subscales which tend to respond more, like burning (superficial) and subscales which do not respond to treatment, like pressing (deep). The patient has to be informed before qualification for the treatment that some symptoms might aggravate after surgery (paresthesia and dysesthesia) (8, 12).

The pathophysiology of neurogenic pain is intricate and includes alterations of multiple neural networks as stated above. Typically used DBS settings of stimulation frequency for PAG/PVG DBS are lower for pain than used for movement disorders. It is believed that lower frequencies (<50 Hz) cause analgesia and higher frequencies (>70 Hz) cause hyperalgesia. Typical settings of PAG/PVG DBS include low frequency (<50 Hz) with a wide spectrum of voltage 0.6–7 V and pulse width spectrum from 60 to 120 μs (19, 21). In the analyzed group, the initial settings were set for: monopolar stimulation (case positive, contact 1 and 2 negative) with amplitude 1 V, frequency 50 Hz, and pulse width 60 μs. At the follow-up visits, depending on clinical response the amplitude was gradually increased up to 5 V (14–16).

There are inherent surgical risks related to DBS. The mortality rate related to DBS is less than 0.4% (pulmonary embolism, myocardial infarction). Intracranial bleeding and intracerebral hemorrhage (1–2%) can cause the most serious complications. Thromboembolic complications, urinary infection and pneumonia are reported in less than 2% of patients. Typical adverse effects of the stimulation of PAG/PVG include: eye bobbing, eye deviation (spread to the superior colliculus and oculomotor nerve) and anxiety. Nausea and diaphoresis are the most common autonomic side effects observed especially with higher voltages. The positive effect of stimulation is frequently preceded by not precisely defined warmth/cold sensation or contralateral to the stimulation paresthesias. Possible adverse effects related to stimulation can be eliminated by changing the settings of the stimulation. Stimulation-related seizures are more frequently observed among patients with motor cortex stimulation (4, 22). Complications related to the implant are more frequent and include: lead migration and fracture (2–3%) and infection (3–8%) (5, 8, 26). At the 24-month follow-up in the analyzed group, no surgery- or implant-related complications were recorded. The majority of the patients reported warmth that appeared predominantly contralateral to the implanted PAG/PVG- mostly in the face, trunk or upper extremity. The feeling of warmth had a tendency to fade away within minutes after changing the settings of the stimulation. Observed eye deviation had a tendency to appear at higher voltages. Those were eliminated by the instant change of settings of the DBS. The mechanism of those adverse effects remains clear. The time frame of appearance and disappearance of those sensations related to the stimulation indicate that most probable mechanism is direct stimulation of surrounding PAG/PVG structures. The role of brain blood flow variations or alterations of endogenous opioid levels related to DBS is less probable. Less frequently patients complained of persistent paresthesia or dysesthesia after changing of the DBS settings. Increased by 10% subscore 5 of NPSI (paresthesia/ dysesthesia) at the 24-month follow-up might be identified as an adverse effect of PAG/PVG DBS even though the paresthesia did not vanish after changing of the DBS settings.

## Conclusion

PAG/PVG DBS is an effective and safe method of treatment of medically refractory neuropathic facial pain. NRS is an easy-to-apply scale for pain evaluation. The effectiveness of the treatment tends to decrease at 2 years of follow-up. The application of NPSI helps to differentiate the type of pain that might respond the best to PAG/PVG DBS. The pain phenotypes which tend to respond the best to PAG/PVG are burning and superficial pain (subscore 1 of NPSI). PAG/PVG is least effective for subscore 2 of NPSI (pressing, deep pain). Symptoms like paresthesia and dysesthesia (question number 11 and 12- subscore 5) might increase after PAG/PVG DBS. Those aggravated symptoms tend to persist even when the stimulation is deactivated. Even though this composite effect of PAG/PVG on the complex phenotype of neuropathic facial pain resulted in poorer results measured with NPSI (compared to NRS), the authors believe that both scales should be applied to measure the efficacy of the treatment.

## Data availability statement

The raw data supporting the conclusions of this article will be made available by the authors, without undue reservation.

## Ethics statement

Ethical approval was not required for the studies involving humans because the patients underwent typical neurosurgical treatment under standard regiment. The studies were conducted in accordance with the local legislation and institutional requirements. The participants provided their written informed consent to participate in this study.

## Author contributions

VM prepared the manuscript and collected data. PZ, BK, KS, and HK conducted follow-up, collected data, and participated in surgery. KC analyzed data. TM performed surgery, conducted follow-up, and coordinated the creation of the manuscript. All authors contributed to the article and approved the submitted version.
